# Treatment Strategies for Chronic Pancreatitis (CP)

**DOI:** 10.3390/ph18030311

**Published:** 2025-02-24

**Authors:** Katarzyna Tłustochowicz, Agnieszka Krajewska, Adrianna Kowalik, Ewa Małecka-Wojciesko

**Affiliations:** Department of Digestive Tract Diseases, Medical University of Lodz, 90-153 Lodz, Poland; katarzyna.tlustochowicz@stud.umed.lodz.pl (K.T.); adrianna.kulis@student.umed.lodz.pl (A.K.)

**Keywords:** chronic pancreatitis, autoimmune pancreatitis, treatment, management, rituximab, nutrition

## Abstract

Chronic pancreatitis (CP) and autoimmune pancreatitis (AIP) are diseases with overlapping features, both requiring complex management strategies. CP is characterized by pancreatic exocrine insufficiency (PEI) and pain, with treatment focused on symptom relief through pancreatic enzyme replacement therapy (PERT), pain control, and lifestyle and nutritional changes. However, the standard therapy does not address the underlying inflammation or fibrosis, which drives disease progression. AIP, on the other hand, presents with obstructive jaundice and fibrosis and is classified into two subtypes: Type 1 (AIP-1), linked to IgG4-related disease, and Type 2 (AIP-2), associated with inflammatory bowel disease. Treatment for AIP typically involves oral steroids. Immunomodulators and rituximab are used for recurrent or refractory cases. Novel therapies targeting the inflammation and fibrotic components of CP are being explored. A multidisciplinary approach is essential to optimize care and improve patients’ outcomes.

## 1. Chronic Pancreatitis (CP)

Chronic pancreatitis (CP) is a long-term inflammatory condition that leads to fibrosis and irreversible damage to pancreatic tissue. It is a progressive condition with no treatment currently available to reverse its course [1]. Advances in medical technology are continually improving diagnostic and treatment methods, but despite these developments, managing CP remains challenging [2]. CP encompasses a broad spectrum of exocrine pancreatic disorders, including calcifying, obstructive, and steroid-responsive forms [3]. The Toxic-metabolic, Idiopathic, Genetic, Autoimmune, Recurrent, and severe acute pancreatitis and Obstructive (TIGAR-O) Pancreatitis Risk/Etiology Checklist (TIGAR-O_V1) is a broad classification system that lists the major risk factors and etiologies of recurrent acute pancreatitis, chronic pancreatitis, and overlapping pancreatic disorders. New discoveries and progressive concepts since the 2001 TIGAR-O list required an update in 2019 to TIGAR-O_V2 with both a short (S) and long (L) form [4]. The updated system is structured as a hierarchical checklist, enabling healthcare professionals to efficiently record and monitor specific elements. The most common cause of CP is chronic alcohol abuse [5]. Other contributing factors include genetic predisposition, cigarette smoking, recurrent acute pancreatitis, and metabolic disturbances [6]. The prevalence of hereditary pancreatitis is rising. The most commonly mutated genes in CP include the cationic trypsinogen gene (*PRSS1*), the pancreatic secretory trypsin inhibitor gene (*SPINK1*), the cystic fibrosis transmembrane conductance regulator gene (*CFTR*), the chymotrypsinogen gene (*CTRC*), and the carboxy peptidase A1 gene (*CPA1*) [7,8,9,10,11]. One of the most common consequences of chronic pancreatitis is pancreatic exocrine insufficiency (PEI), which results in symptoms such as maldigestion, malabsorption of food and nutrients, and over time, malnutrition [12,13]. Malnutrition is primarily caused by reduced pancreatic enzyme secretion and decreased caloric intake [14]. The primary clinical sign of PEI is steatorrhea, particularly in advanced stages, although its absence does not exclude the disease. Osteopenia and osteoporosis are the consequences of malnutrition, especially in patients with CP, who have impaired vitamin D absorption and intake, which can result in an increased fracture risk [15]. To assess nutritional status and detect malnutrition, patients with CP should undergo body mass index (BMI) screening using tools such as the Malnutrition Universal Screening Tool (MUST) for community settings, which uses five steps and assesses risk based on BMI, unplanned weight loss, and the impact of acute disease. For hospitalized patients, the Nutritional Risk Screening (NRS-2002) is recommended, as it evaluates nutritional status, disease severity, and age [16]. Additionally, screening for deficiencies in fat-soluble vitamins (A, D, E, and K), as well as zinc, magnesium, and glycated hemoglobin (HbA1c), is recommended. All manifestations of the disease contribute to a reduced quality of life for these patients. The most serious long-term complication of CP is an 8-fold increased risk of developing pancreatic cancer [17].

A definitive diagnosis of chronic pancreatitis requires a combination of clinical history, imaging, and functional testing rather than reliance on any single diagnostic method alone. In advanced stages, diagnosis is often straightforward due to typical symptoms and characteristic findings, such as a dilated pancreatic duct, pancreatic atrophy, and pancreatic calcifications, which can be detected using computed tomography (CT), magnetic resonance imaging (MRI), and endoscopic ultrasound (EUS).

The Cambridge criteria were developed to standardize the interpretation of pancreatic imaging findings, initially based on endoscopic retrograde cholangiopancreatography (ERCP), ultrasound (US), and CT. However, ERCP is no longer used for diagnosing chronic pancreatitis and is now primarily reserved for interventional therapeutic procedures. US and CT classify chronic pancreatitis based on various features, including main pancreatic duct enlargement (>4 mm), gland enlargement (up to twice the normal size), small cavities (<10 mm), irregular ducts, focal acute pancreatitis, parenchymal heterogeneity, increased duct wall echogenicity, and irregular head/body contour. These findings are categorized as equivocal (one feature present), mild to moderate (two or more features), or severe, characterized by large cavities (>10 mm, significant gland enlargement (>2× normal size), intraductal filling defects or calculi, duct obstruction or structures, gross irregularity, contiguous organ invasion [18].

In 2019, a modified Cambridge classification was proposed based on contrast-enhanced CT scans of 158 well-phenotyped patients with chronic pancreatitis. The revision excluded subjective features such as gland heterogeneity and irregular head/body contour while incorporating both ductal and parenchymal characteristics, including pancreatic atrophy, with severity scoring. Morphological severity (equivocal, mild, moderate, severe) and subtypes (non-calcific and non-obstructive, calcific but non-obstructive, obstructive with or without calcifications) were defined using a combination of these findings. This modification enables a more objective stratification of the disease stage and facilitates longitudinal assessment in individual patients [19].

The Rosemont criteria were developed to improve the interpretation of EUS findings in CP. Based on the combination of major and minor features, CP is classified as consistent, probable, or indeterminate. The major criteria were divided into Type A and Type B. Major A criteria include hyperechoic foci with shadowing and main pancreatic duct calculi, while Major B criteria encompass lobularity with a honeycomb pattern. The minor criteria include a dilated pancreatic duct (>3.5 mm), pancreatic cysts, an irregular pancreatic duct, dilated side branches (>1 mm), a hyperechoic duct wall, strands, non-shadowing hyperechoic foci, and lobularity with noncontiguous lobules [20].

## 2. Chronic Pancreatitis Management: General Recommendations About the Lifestyle

A balanced and healthy diet without fat restriction is recommended for patients with CP, according to clinical guidelines [14]. Moreover, a diet high in protein is suggested for patients with poor nutritional status [14]. A high-protein diet in patients with renal disease needs to be administered with caution and renal parameter monitoring. A diet low to moderate in fiber is recommended, as high fiber intake can diminish the effectiveness of supplemental enzyme therapy and contribute to malabsorption [14,21]. Most guidelines recommend the cessation of alcohol in CP patients [22], although whether alcohol cessation alters the natural history of the disease is still unknown [23]. Several case series have suggested that discontinuing alcohol intake alleviates pain in CP but does not necessarily alter the progression of the disease [22,24]. Smoking cessation is advised for all CP patients, as smoking is an independent risk factor for the development of CP [25,26]. Case series have shown that smoking cessation at the time of CP diagnosis may reduce the progression of pancreatic calcifications [27].

## 3. Pancreatic Enzyme Replacement Therapy (PERT) in Pancreatic Exocrine Insufficiency (PEI)

The newest European guidelines for the diagnosis and treatment of pancreatic exocrine insufficiency (PEI) from 2024 recommend that pancreatic enzyme replacement therapy (PERT) is indicated for all patients with confirmed PEI [28]. Symptoms and nutritional deficiencies associated with PEI are nonspecific. Therefore, a combined evaluation of symptoms, nutritional status, and pancreatic function within the relevant clinical context is recommended for diagnosing PEI in clinical practice. The nutritional status of patients with PEI is primarily assessed through anthropometric measurements and blood parameters of malnutrition. Single assessments of parameters such as body weight, BMI, weight loss, lean body mass, and muscle mass are insufficiently sensitive for diagnosing PEI. Instead, serial measurements are more reliable in detecting trends and changes. Limited evidence suggests that serum levels of fat-soluble vitamins (A, D, E, and K), trace elements (e.g., magnesium, selenium, and zinc), and plasma proteins, including retinol-binding protein, albumin, and prealbumin, may have diagnostic value in identifying PEI [28]. However, due to the limited quality of available evidence, no strong recommendations can be made regarding their routine use in clinical practice. Non-invasive tests such as the Fecal Elastase-1 Test (FE-1) and 13C-MTG (Mixed Triglyceride) Breath Test are recommended for assessing pancreatic exocrine function [29]. Fecal elastase-1 quantification is the most common method for assessing pancreatic enzyme secretion, as elastase is a stable, pancreas-specific enzyme [19]. The limitation of the FE-1 test is that it is insensitive to mild PEI [22]. A fecal elastase level below 100 μg/g of stool strongly indicates the presence of PEI, while levels ranging from 100 to 200 μg/g are considered inconclusive for diagnosing PEI [30].

Pancreatic enzyme replacement therapy (PERT) is the current standard treatment of pancreatic exocrine insufficiency [31]. PERT preparations are recommended as the first-line treatment for PEI, including pancreatin (Creon, Zenpep) [28]. The initial doses of PERT primarily depend on the severity of PEI, the patient’s age (adult or child), and the meal’s fat content and are based on lipase activity [28]. Typical minimum doses in adults include 40,000–50,000 units of lipase per meal and half of that dose of 20,000–25,000 units of lipase with snacks [28]. However, patients with severe PEI, such as those who have undergone pancreaticoduodenectomy, have been reported to benefit from a higher initial dose of PERT [28]. Treatment of PEI is most effective when taken with meals or shortly after meals to ensure proper mixing with chyme in the stomach [29].

Numerous studies have shown significant benefits from using PERT, including improvement in body weight and BMI, reduced stool frequency, and overall improvement in quality of life [1,15]. Furthermore, clinical studies indicate that PERT is safe, effective, and well tolerated [32,33]. PERT is not associated with major adverse effects; the main adverse effects of PERT are gastrointestinal disorders such as diarrhea, nausea, flatulence, and abdominal pain. Allergic skin reactions (rash, urticaria) can also occur [1,34]. Patients who do not respond or only partially respond to PERT should be carefully evaluated for issues such as nonadherence or improper administration of PERT [28].

## 4. Fat-Soluble VITAMIN Replacement

CP patients are unable to absorb fat appropriately, which causes an inability to efficiently absorb fat-soluble vitamins. As a result, they are likely to develop various nutrient deficiencies, including fat-soluble vitamins (A, D, E, and K) as well as vitamin B_12_, folate, iron, calcium, zinc, selenium, and magnesium [35,36]. The symptoms of vitamin deficiencies are as follows: vitamin K—petechial hemorrhages, vitamin E—peripheral neuropathy, vitamin A—night blindness, and vitamin D—muscle crumps, osteomalacia, and osteoporosis [16]. Screening for vitamin and mineral deficiencies is important at the time of diagnosis and annually thereafter, depending on the patient’s clinical condition [31]. Key blood parameters indicative of malnutrition, such as prealbumin, retinol-binding protein, 25-OH cholecalciferol (vitamin D), and essential minerals and trace elements (including serum iron, zinc, and magnesium), should be assessed [16]. If vitamin deficiencies occur, routine supplementation is recommended [30]. Reedy et al., in a randomized controlled trial (RCT), assessed that a single intramuscular injection of 600,000 IU (15,000 μg) of cholecalciferol is more effective in achieving vitamin D sufficiency over 6 months compared with 300,000 IU (7500 μg) of vitamin D3. No patients developed hypervitaminosis D or hypercalcemia, suggesting that both doses were safe [37]. In addition, calcipotriol, a synthetic analog of vitamin D3, is known to have a protective anti-inflammatory effect against pancreatitis [38,39].

## 5. Pain Management in Chronic Pancreatitis

Abdominal pain is the most frequent symptom of chronic pancreatitis (CP), significantly worsening patients’ quality of life [2]. Treatment options include pharmacological agents, endoscopy, and surgery. Pharmacological pain management in CP is based on the analgesic ladder provided by the World Health Organization (WHO) [16]. According to this approach, first-line therapy includes non-opioid analgesics. Acetaminophen is the preferred analgesic due to its safety profile [16]. However, it is worth mentioning that its use requires caution, as an overdose is associated with a significant risk of hepatotoxicity. The maximum recommended dose for adults is 4 g per day. Non-steroidal anti-inflammatory drugs should be avoided because their gastrointestinal toxicity may aggravate abdominal pain [16]. Oral weak opioids are a secondary option when first-line drugs are ineffective [40]. Among weak opioids, tramadol is preferred [16]. It has been shown that tramadol is superior to morphine in patients with CP, providing similar levels of analgesia but with fewer gastrointestinal side effects [16]. Third-line therapy includes strong opioids such as morphine [16]. The smallest effective doses of opioids should be used, and the drug should be administered orally to prevent dose escalation and reduce the risk of addiction [16]. Another therapy option in managing CP pain is adjuvant analgesics, such as pregabalin, which are a heterogeneous group of drugs primarily developed for other indications. In a randomized, controlled trial, pregabalin significantly reduced pain compared to placebo [41]. The study involved 64 patients with CP-related pain who received increasing oral doses of either pregabalin or placebo over three weeks. The initial dose of pregabalin was 75 mg twice daily, increasing to 150 mg twice daily after 3 days and to 300 mg twice daily after 1 week for the remainder of the study. Pain level was evaluated using a visual analog scale recorded in a pain diary, while patient-related health status was measured with the Patients’ Global Impression of Change (PGIC) score. The results indicated that pregabalin provided significantly greater pain relief, with a higher percentage of patients benefiting compared to placebo (36% vs. 24%). A similar improvement was observed in health status, with 44% of patients reporting benefits compared to 21% with placebo [41].

## 6. Management of Type 3 Diabetes in Chronic Pancreatitis

Type 3c diabetes, also known as pancreatogenic diabetes, occurs due to damage to the pancreatic islets of Langerhans from primary pancreatic disorders [42]. Chronic pancreatitis is the most common cause of Type 3c diabetes [42]. This type of diabetes is often misdiagnosed as Type 2 diabetes but differs in its cause, clinical presentation, and treatment. Patients with Type 3c diabetes face challenges such as diarrhea and malabsorption, complicating blood glucose management [42]. It often affects individuals with a history of alcoholism, making poor adherence to the treatment and complicating further care. Treatment primarily involves insulin therapy to manage blood glucose levels and pancreatic enzyme replacement to address exocrine insufficiency [42]. Insulin therapy should be used with caution because of the risk of hypoglycemia due to the impaired glucagon function inherent to Type 3c diabetes [42]. A multidisciplinary approach and patient education are crucial for effective management and improved outcomes of diabetes Type 3c. 

## 7. Novel Possible Therapeutics in Chronic Pancreatitis

Pancreatic fibrosis is caused by the activation of pancreatic stellate cells (PSCs) that proliferate and produce extracellular matrix components, such as type I collagen, and express cytokines and chemokines [43]. Fibrosis in CP leads to the atrophy of acinar structures and the deposition of extracellular matrix (ECM), which impairs both the exocrine and endocrine functions of the pancreas [44]. PSCs undergo morphological transformations linked to the secretion of soluble factors, which enhance the inflammatory process. These changes include the expression of α-SMA, increased proliferation, production of extracellular matrix proteins such as type I and III collagen, generation of cytokines and chemokines, and expression of adhesion molecules like ICAM-1 [45]. Unfortunately, the current standard treatments for CP do not address fibrosis prevention [46]. Emerging and experimental therapies focus on reducing fibrosis and inflammation. Each new therapy is directed to target specific pathways involved in the pathogenesis of CP, aiming to limit disease progression and improve patients’ quality of life. Most of the new therapies’ targets include suppressing the proliferation and activation of pancreatic stellate cells (PSCs), inhibiting M1 and M2 macrophage polarization, and preventing macrophage infiltration into the pancreas [47,48]. While many strategies remain in the preclinical stage using animal models, several strategies have progressed into clinical trials [49,50]. Ciofoaia et al. conducted a Phase 1 open-label safety study on eight patients with chronic pancreatitis (CP) experiencing moderate to severe abdominal pain [50]. Patients aged 21 to 68 years were treated with the cholecystokinin (CCK) receptor antagonist proglumide at an oral dose of 1200 mg per day for 12 weeks [50]. CCK is a hormone that stimulates pancreatic fibrosis. Proglumide is a CCK receptor antagonist, potentially reducing fibrosis by blocking CCK’s effects [50]. MiR-185-5p, miR-346-5p, and miR-378-3p were measured because they are known to inhibit fibrosis in stellate cells by blocking the translation of pro-fibrotic genes and inducing the degradation of target mRNAs. MiR-122-5p was monitored as it indicates inflammation levels [50]. The treatment was found to be safe and well tolerated, with no serious side effects reported. Two patients experienced nausea and diarrhea. Blood hematology and chemistry test results showed no significant changes compared to baseline values, with serum chloride being the only chemistry value that increased at week 12 [50]. Blood microRNA biomarker analysis revealed a statistically significant increase in serum levels of miR-185-5p (1 ± 0 to 1.53 ± 0.22), miR-346-5p (1 ± 0 to 1.34 ± 0.12), and miR-378-3p (1 ± 0 to 1.52 ± 0.19), correlating with fibrosis inhibition [50]. Additionally, a decrease in serum levels of miR-122-5p (1 ± 0 to 0.55 ± 0.074) indicated reduced inflammation [50]. By week 12 of proglumide therapy, pain scores significantly improved compared to pre-treatment levels [50]. Ciofoaia et al. concluded that proglumide therapy, at a dose of 1200 mg per day, is safe and well tolerated and may have potential in the treatment of CP [50].

## 8. Autoimmune Pancreatitis

Autoimmune pancreatitis (AIP) is a specific form of pancreatitis characterized by obstructive jaundice with or without pancreatic masses and is marked by lymphoplasmacytic infiltration, fibrosis, and a significant response to steroid treatment [51]. Two subtypes of AIP have been identified: Type 1 AIP (AIP-1) and type 2 AIP (AIP-2).

AIP-1 is a pancreatic manifestation of the systemic fibro-inflammatory condition, IgG4-related disease (IgG4-RD), involving multiple organs [52]. Histologically, it shows lymphoplasmacytic sclerosing pancreatitis (LPSP) [53,54]. The pathogenesis of AIP-1 is not fully understood but may involve innate immunity. The overexpression of specific toll-like receptors (TLRs) in the pancreas and salivary glands of patients with AIP and IgG4-RD suggests a role for the innate immune system, while plasmacytoid dendritic cells (pDCs) and their unregulated production of IFN-α can be associated with AIP [55,56]. Although AIP-1 is usually painless, some patients may experience abdominal pain, weight loss, malaise, and, in rare cases, acute pancreatitis [57,58].

AIP-2 is limited to the pancreas and linked to inflammatory bowel disease, especially ulcerative colitis. Symptoms include abdominal pain, acute pancreatitis, and painless jaundice [59,60]. The pathogenesis of AIP-2 is unknown, but the Th-17 cells may play a role in infiltrating the periductal pancreatic tissue and releasing inflammatory cytokines [61,62]. AIP-2 may also be linked with PKHD1 and with multiple endocrine neoplasia 1 (MEN1) [63].

The International Consensus Diagnostic Criteria for the treatment of autoimmune pancreatitis stated that therapy should be introduced to symptomatic patients with abdominal or back pain, fever, obstructive jaundice, or other organ involvement [64]. In addition, the therapy may be introduced in asymptomatic patients with a persistent pancreatic mass on imaging or in patients with abnormal liver test results [64]. Approximately 10–25% of AIP-1 cases show spontaneous resolution of symptoms without medical treatment [53]. Steroids are the first-line therapy for patients with active AIP [64]. They inhibit the expression of many proinflammatory cytokines involved in AIP as well as inhibit dendritic cell maturation and downstream signal transduction of TLRs; overexpression in the pancreas plays a role in the innate immune system and development of AIP [64,65,66]. The initial dose of oral prednisone should be 0.6–0.8 mg/kg per day (typically 30–40 mg/day) for one month, but the dose can be adjusted. This adjustment is based on body weight in the case of particularly aggressive disease (the initial dose can be above 40 mg/day) or in the case of elderly patients with very mild clinical symptoms (the dose can be under 20 mg/day) [65]. The assessment of response to treatment should be considered after 2–4 weeks of treatment with the assessment of biochemical and morphological markers such as liver enzymes, IgG or IgG4 levels, and repeated imaging findings [65]. Afterward, the prednisone dose should be tapered by 5 mg every 2 weeks and suspended after 3–6 months of therapy based on clinical changes [64,65]. Most AIP-1 patients respond well to steroid treatment with resolution of clinical symptoms, normalization of IgG4 serum levels, and disappearance of typical AIP features on imaging [67]. Patients with incomplete response to treatment or with relapse of symptoms may require maintenance therapy. No relevant data are available to recommend maintaining low-dose therapy for a few months, although several experts recommend maintaining oral glucocorticoids with a dose of 2.5–10 mg of prednisolone per day for 12 months [65]. Yoon et al. 2021 conducted a systematic literature review and meta-analysis of 40 studies examining the relapse of AIP [68]. Relapse was defined based on pancreatic radiologic manifestations and biochemical abnormalities, while isolated elevation of serum IgG4 was not considered indicative of relapse [68]. This review analyzed 3948 patients with AIP-1 and relapse of the disease across various durations of glucocorticoid therapy. The results showed a decrease in the relapse rate of type 1 AIP with extended glucocorticoid therapy: 47% with 6 months of therapy, 44% with 6–12 months, 34% with 12–36 months, and 27% with over 36 months of therapy [68].

The immunomodulators (IM) such as thiopurines (azathioprine and 6-mercaptopurine) can be used in the management of AIP patients [65]. Adding immunosuppressive agents should be considered in patients who experience a relapse during the first 3 months of standard steroid therapy (steroid tapering or discontinuation), as well as in patients with a high risk of relapse [65]. Thiopurines, including 6-mercaptopurine (6-MP) and azathioprine (AZA), are purine antimetabolites that interfere with cellular proliferation, modulating both innate and adaptive immunity [69]. According to a literature review, azathioprine is the most used (85%) immunosuppressant [70]. The suggested dose of oral azathioprine is 2–2.5 mg/kg body weight, with close clinical and laboratory monitoring [71]. Thiopurines are associated with serious side effects such as myelosuppression, hepatotoxicity, and gastrointestinal intolerance (nausea and vomiting) and should be used with caution [65].

Chloroquine (CQ) and hydroxychloroquine (HCQ) are drugs with therapeutic potential in managing AIP [72]. They inhibit endosomal toll-like receptors (TLR7 and TLR9), which play a crucial role in triggering inflammatory responses and the production of IFN-1, promoting inflammation and fibrosis characteristic of AIP [72]. Although chloroquine and hydroxychloroquine are effectively used in other autoimmune diseases, such as systemic lupus erythematosus (SLE) [72], their mechanism of action, targeting inflammatory pathways, suggests it could become a valuable addition to AIP treatment strategies [72]. M. Li et al. investigated the role of HCQ in the treatment of AIP-2 [73]. In this study, researchers analyzed pancreatic tissue samples from patients with type 2 autoimmune pancreatitis (AIP), pancreatic cancer (PDAC), and healthy controls (HC), assessing inflammatory responses and the effects of hydroxychloroquine (HCQ) on cytokine production, neutrophil migration, and NET formation. Hydroxychloroquine functions as a TLR9 antagonist, a key factor in the inflammatory signaling pathways within pancreatic ductal epithelial cells (HPDEC) [73]. By inhibiting neutrophil extracellular traps (NETs)-triggered TLR9 signaling, HCQ effectively reduces the expression of proinflammatory factors such as IL-1β, IL-6, and IL-8 in a dose-dependent manner [73]. This selective action is particularly evident in cells with overexpressed TLR9, where HCQ significantly diminishes the production of proinflammatory cytokines and chemokines while having minimal impact on control cells [73]. Moreover, HCQ markedly decreases the migration and chemotaxis of neutrophils towards activated HPDEC and inhibits the formation of NETs. These effects highlight HCQ’s potential use as a therapeutic agent for managing AIP-2 by specifically targeting TLR9-related pathways and mitigating the inflammatory response [73].

Rituximab (RTX) is a monoclonal antibody directed against the B-cell-specific antigen CD-20 and is the second-line therapeutic choice for acute AIP-1 [65,74]. There is a compelling pathophysiological basis for using rituximab in IgG4-related disease, as it effectively targets and depletes CD20+ B-cell precursors responsible for disease-specific clonal plasmablasts [65]. RTX therapy should be considered if treatment with high-dose glucocorticoids is ineffective or not tolerated [65]. The dosing protocol for RTX is 375 mg/m^2^ body surface area, administered weekly for 4 weeks, followed by infusions every 2–3 months, or as two 1000 mg infusions 15 days apart every 6 months [65,71]. Hart et al. conducted a study involving patients with immunomodulator resistance/intolerance or steroid intolerance, where RTX therapy was introduced [75]. The dosing protocol for RTX consisted of 375 mg/m^2^ body surface area administered intravenously once weekly for 4 weeks, followed by repeat infusions every 2 to 3 months for a total duration of 24 months (8 infusions in total) [75]. Ten out of twelve patients (83%) achieved complete remission, with eight having complete radiographic remission documented and the other two patients having either improved or stable biliary disease. Additionally, four patients had complete normalization of IGG4 level. During the maintenance phase, with a median follow-up of 10.6 months, no disease relapse was observed. However, one patient developed weight loss and symptoms of steatorrhea over 2 years after completing the 24-month RTX course [75].

The potential use of biologic medications in the treatment of AIP-2 is discussed [60]. In 2020, Lorenzo et al. described a case of a patient with AIP-2 and with bad tolerance and resistance to steroids. The patients received an induction of three adalimumab doses without subsequent maintenance therapy [76]. Steroid treatment was discontinued a few weeks after the third adalimumab dose. The patient experienced no disease recurrence during the 11-month follow-up period [76]. Lauri et al. presented two cases of patients diagnosed with autoimmune pancreatitis type 2 (AIP-2) and inflammatory bowel disease (IBD) who were treated with ustekinumab, a monoclonal antibody targeting the shared p40 subunit of interleukins (IL)-12 and IL-23. Both patients received the standard dosing regimen for IBD [77]. In the first case, endoscopic ultrasound (EUS) after six months of therapy demonstrated a stable chronic pancreatitis (CP) profile. In the second case, EUS performed six months post-treatment revealed no signs of swollen or edematous parenchyma; instead, only a few hyperechoic spots were noted, representing a significant improvement compared to the pre-treatment findings, which were characterized by a swollen and hypoechoic appearance [77].

## 9. Conclusions

Chronic pancreatitis (CP) is a complex disease that impairs quality of life (QoL) and increases the risk of malnutrition-related complications. Diagnosing PEI requires assessing symptoms, nutritional status, and pancreatic function. All patients diagnosed with PEI require treatment, with the first-line recommended treatment being pancreatic enzyme preparations, especially pancreatin. PERT improves body weight, nutritional status, symptoms, and quality of life. Unfortunately, the PERT approach for chronic pancreatitis is primarily based on symptom relief and does not focus on reducing inflammation or minimizing pancreatic fibrosis. Pain management in CP is based on the analgesic ladder provided by the WHO, with non-opioid analgesics being the first-line treatment, while adjuvant analgesics such as pregabalin can be added to enhance pain control. This approach aims to tailor therapy to the severity and nature of the pain while minimizing the reliance on opioids. AIP is a rare and complex disease that typically shows a remarkable response to corticosteroid therapy. However, in cases of intolerance or relapse on steroids, alternative therapies such as rituximab and other immunomodulators have been effectively utilized. AIP is characterized by a multitude of mechanisms contributing to its etiopathogenesis, suggesting the need for continued scientific efforts to identify new and effective therapeutic options. Emerging studies on the use of biologics, including adalimumab and ustekinumab, are particularly promising for the management of refractory or relapsing cases of AIP.
